# Multiple long-term, experimentally-evolved populations of *Escherichia coli* acquire dependence upon citrate as an iron chelator for optimal growth on glucose

**DOI:** 10.1186/1471-2148-12-151

**Published:** 2012-08-21

**Authors:** Nicholas Leiby, William R Harcombe, Christopher J Marx

**Affiliations:** 1 Systems Biology Graduate Program, Harvard University, Cambridge, MA, USA; 2 Department of Organismic and Evolutionary Biology, Harvard University, Cambridge, MA, 02138, USA; 3 Faculty of Arts and Sciences Center for Systems Biology, Harvard University, Cambridge, MA, USA

**Keywords:** Tradeoffs, Specialization, Iron-limitation, Citrate

## Abstract

**Background:**

Specialization for ecological niches is a balance of evolutionary adaptation and its accompanying tradeoffs. Here we focus on the Lenski Long-Term Evolution Experiment, which has maintained cultures of *Escherichia coli* in the same defined seasonal environment for 50,000 generations. Over this time, much adaptation and specialization to the environment has occurred. The presence of citrate in the growth media selected one lineage to gain the novel ability to utilize citrate as a carbon source after 31,000 generations. Here we test whether other strains have specialized to rely on citrate after 50,000 generations.

**Results:**

We show that in addition to the citrate-catabolizing strain, three other lineages evolving in parallel have acquired a dependence on citrate for optimal growth on glucose. None of these strains were stimulated indirectly by the sodium present in disodium citrate, nor exhibited even partial utilization of citrate as a carbon source. Instead, all three of these citrate-stimulated populations appear to rely on it as a chelator of iron.

**Conclusions:**

The strains we examine here have evolved specialization to their environment through apparent loss of function. Our results are most consistent with the accumulation of mutations in iron transport genes that were obviated by abundant citrate. The results present another example where a subtle decision in the design of an evolution experiment led to unexpected evolutionary outcomes.

## Background

Evolutionary adaptation to a new environment leads to changes that improve function and increase fitness, but it may also result in the deterioration of functions not needed in that selective environment. These declines in function that accompany adaptation – tradeoffs – are often considered inevitable costs or constraints of adaptation [1]. Tradeoffs can either result from antagonistic pleiotropy, when adaptive mutations in the selective environment are deleterious elsewhere, or from the accumulation of mutations that are neutral in the selective environment. A consequence of these tradeoffs is that they prevent ‘Darwinian demons’: single super-genotypes that are optimally fit across a spectrum of environments [2]. Instead, there are usually ‘generalist’ organisms that perform adequately in a variety of environments that can coexist with ‘specialist’ organisms that occupy smaller niches but with greater effectiveness.

Critical to the emergence of specialization is the constancy of an environment [3]. By occupying the same environment for an extended period of time, the advantage of maintaining fitness in alternate environments is not realized. Organisms may thus be expected to become increasingly specialized to their current, static environment. The most obvious form of specialization is a decline in the ability to utilize resources or exist in conditions that were not experienced during adaptation. Many such examples have been observed during laboratory evolution, whereby organisms have evolved to narrow or shift their range of preferred temperatures [4], carbon sources [5,6], host organisms [7], or even laboratory water supply [8]. Alternatively, organisms can lose the ability to grow well in the absence of a resource that is currently available. This has been observed, in particular, for microbes infecting hosts. Examples range from the long-term, dramatic losses by intracellular symbionts [9] to the rapid emergence of auxotrophy during passage through a mouse gut [10].

One of the most prominent examples of prolonged adaptation to a single environment is the Lenski Long-Term Evolution Experiment (LTEE) [11]. In this experiment, 12 populations of *Escherichia coli* were founded with either the arabinose-negative strain REL606 (populations A-1 to A-6) or the otherwise isogenic arabinose-positive derivative, REL607 (A + 1 to A+6). These have evolved since 1988 in Davis-Mingioli (DM) minimal media [12] batch cultures containing glucose as a growth substrate. Over 50,000 generations, the fitness of the evolved strains in the evolutionary environment has increased substantially, and appears to continue to do so [13]. In line with the expectation of a generalist-specialist tradeoff, evolving strains have also specialized for aspects of their static environment. Evolved isolates have lower fitness in some alternative environments [4,5,14,15], despite the fact that many such individual mutations can be generally beneficial across environments [16].

Perhaps the most surprising adaptive change to have occurred during the LTEE was the huge increase in fitness of one population due to evolving the ability to metabolize citrate, the “inert” metal chelator present in DM minimal medium. Disodium citrate (which we will hereafter refer to as citrate) was included in the evolutionary growth media as a historical artifact of the media’s original formulation for penicillin enrichment of auxotrophs [12,17]. The common use of citrate in minimal media formulations owes to its ability to serve as a chelator of Fe (III). Indeed, no direct addition of iron is made to DM minimal media. Given the quite low level of glucose used in the LTEE (25 mg/L = 0.14 mM), substantially more citrate was present (1.7 mM) than glucose. A diagnostic trait of most *E. coli* strains is that they can only grow on citrate anaerobically, whereas *Salmonella*, for example, can grow on it aerobically. This inability of the ancestral strain to grow on citrate during the aerobic conditions of the LTEE therefore initially rendered this organic acid an unavailable secondary resource. Incredibly, after 31,000 generations – and in just one of the 12 replicate populations (A-3) – the ability emerged to utilize citrate as a sole carbon source during aerobic growth [18]. Interestingly, this was not even the first time *E. coli* has acquired the ability to aerobically utilize citrate. Cit^+^* E. coli * K12 had previously been observed to arise spontaneously [19], and high expression. Plastids has been shown to confer aerobic citrate growth upon *E. coli* B [20].

Besides the remarkable story of novel aerobic utilization of citrate, it is unclear whether any of the LTEE lines may have changed in terms of use of citrate as an iron chelator. Bacteria commonly have multiple acquisition systems for various metals, particularly iron, which is required for aerobic respiration due to the hemes found in cytochromes. The acquisition of iron is particularly challenging for microbes because of the low solubility of the Fe (III) species that is available at neutral pH in oxygenated environments. Consequently, metals are often growth limiting in nature in environments ranging from the open ocean [21] to host infections [22]. In the context of infections, iron is sequestered by high-affinity eukaryotic proteins, and the pathogenesis of many infectious diseases relies on the ability of bacteria to strip iron from their host.

Consistent with the fundamental role that iron plays in microbial growth, and its relative scarcity, microbes have developed an arsenal of techniques to procure it. In *E. coli*, transcriptional regulators sensitive to the intracellular concentrations of iron down-regulate iron uptake genes when supplies are adequate. Under conditions of iron deprivation, these same regulators simultaneously up-regulate iron acquisition systems while down-regulating proteins requiring iron [23,24]. One mechanism to obtain iron is to secrete and reabsorb small molecules called siderophores that chelate extracellular Fe (III), as well as the transporters to utilize them. *E. coli* also has the ability to take up Fe (II), and a transport system to directly capture Fe (III) from host proteins like transferrin or lactoferrin, or from heme [25].

There is precedent for selection acting upon metal acquisition during experimental evolution. Growing *Methylobacterium* at low levels of cobalt repeatedly selected for transposition events upstream of a single cobalt transporter, leading to increased uptake rates [26]. Interestingly, the selective effect of these mutations was dependent upon both the carbon source and the growth rate of cells. Additionally, selection has been observed on metal acquisition as a social trait. Genotypes of *Pseudomonas aeruginosa* that fail to produce siderophores have emerged during adaption in laboratory conditions, as well as over the course of infection in the lungs of cystic fibrosis patients [27,28]. Because excreted siderophores become public goods, in well-mixed environments, non-producers can have a selective advantage over producers [29].

Here we investigated whether other strains from generation 50,000 of the Lenski LTEE have evolved to become dependent on citrate for their performance growing on glucose. There are four non-exclusive hypotheses for the dependence of glucose growth of each population upon citrate. H0: The null hypothesis is that there is no significant stimulation (or perhaps even inhibition), H1: As disodium citrate is the sole source of sodium in the LTEE media, there may be stimulation by sodium ions in a manner independent of citrate itself, H2: Evolved strains use – at least partially – citrate as a growth substrate (e.g., the Cit^+^ A-3 population), and H3: Evolved strains have come to rely upon citrate to chelate iron present in the media.

We found that the ancestors and most of the evolved populations were largely insensitive to the presence of citrate (H0). In contrast, three lineages in addition to the Cit^+^ A-3 population have evolved increased growth rate and yield in the presence of citrate. In no case was this was due to sodium ions (H1). Unlike the Cit^+^ A-3 (H2), however, these lineages neither grew on citrate directly nor incorporated isotopic label from citrate during growth on 100% U-^13^C-glucose. These three lineages were at least partially rescued by the direct addition of Fe (II). These data are consistent with H3: these three populations have evolved to rely on citrate for its original intent, as a chelator of iron. As such, these three populations have evolved a novel dependence upon citrate due to reduction in citrate-independent glucose growth rather than the gain of growth on citrate as a carbon and energy source.

## Results

### Evolved strains acquired dependence on citrate

**Figure 1:**
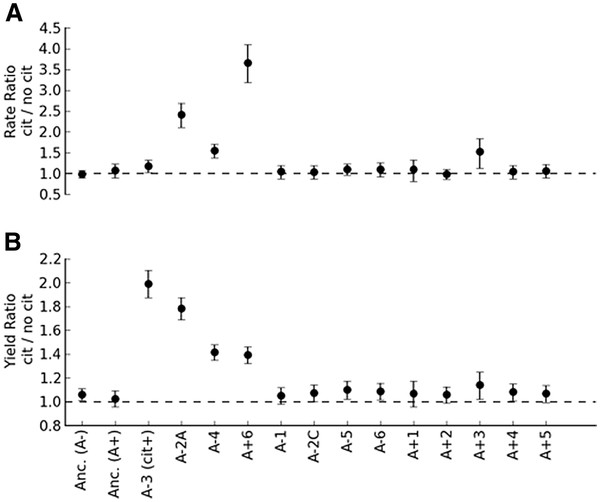
**Dependence of growth on glucose upon the presence of citrate for the ancestors and evolved strains.** The ratio and error for growth parameters was calculated by fitting a log-linear model with the strain:media interaction terms and block as the dependent variables. Unlike the ancestors (first two values), strains from three of the evolved populations were strongly stimulated by citrate in terms of both (**A**) growth rate and (**B**) yield. The remaining nine Cit^-^ lineages, like their ancestors, exhibited little to no dependence upon citrate. We tested both ‘large’ (A-2A) and ‘small’ (A-2C) clones from the A-2 population, as it is known to have a stable, long-term polymorphism. Values represent the mean and 95% confidence intervals for the ratio of growth on glucose with citrate to growth without citrate for a given strain.

In order to test for acquired dependence upon citrate (i.e., rule out H0), we compared growth rate and yield on glucose either with or without citrate for the ancestors and one clonal isolate from each of the 12 populations of the LTEE at 50,000 generations (Figure 1). Growth rate is known to be strongly correlated with fitness in batch cultures in general, and in the LTEE in particular [30]. Yield, while not a component of fitness during batch culture, conveys additional information about cell physiology and was therefore also analyzed. Differences seen in apparent final yield were not explainable by the changed osmolarity due to the addition of citrate to the media; citrate caused no detectable change in optical density (p = 0.95, two-tailed paired T test). Both ‘small’ and ‘large’ clones from the A-2 population were included [31] in the analysis. 

The Cit^+^ A-3 population was seen to grow faster and to a much higher final yield in the presence of citrate, as previously reported [18]. The growth rate of the ancestor REL606 was neither significantly decreased nor enhanced by the addition of citrate (P = 0.71, two-tailed Welch’s T test unless stated otherwise), but there was a very slight, but significant increase in yield (P = 0.0001). Nine of the 13 evolved strains had similarly small increases in rate or yield. While in some cases there were statistically significant increases, the effect was small. For the remaining three strains, however, the increase was much greater for both rate and yield. In the presence of citrate, A-2A, A-4 and A+6 all experienced significant increases in both rate and yield (Respectively, rate: P = 1.6x10^-7^, 5.3x10^-13^, and 3.1x10^-12^, yield P = 1.6x10^-14^, 1.7x10^-7^, and 1.8x10^-9^). Indeed, these effects were quite substantial: the growth rate of A+6 with citrate was 3.75 times faster than growth without citrate, and the final yield of A-2A increased by 80% by the addition of citrate. In comparison, at the 1 mM concentration of glucose used, the Cit^+^ A-3 strain grew 1.2 times faster and increased in final yield by 100% with the addition of citrate. To explore the nature of the citrate dependence, we focused on these three strains for which the effect was greatest, and omitted strains for which the effect was marginal (as well as the Cit^+^ A-3).

### Disodium citrate dependence is not driven by sodium

**Figure 2:**
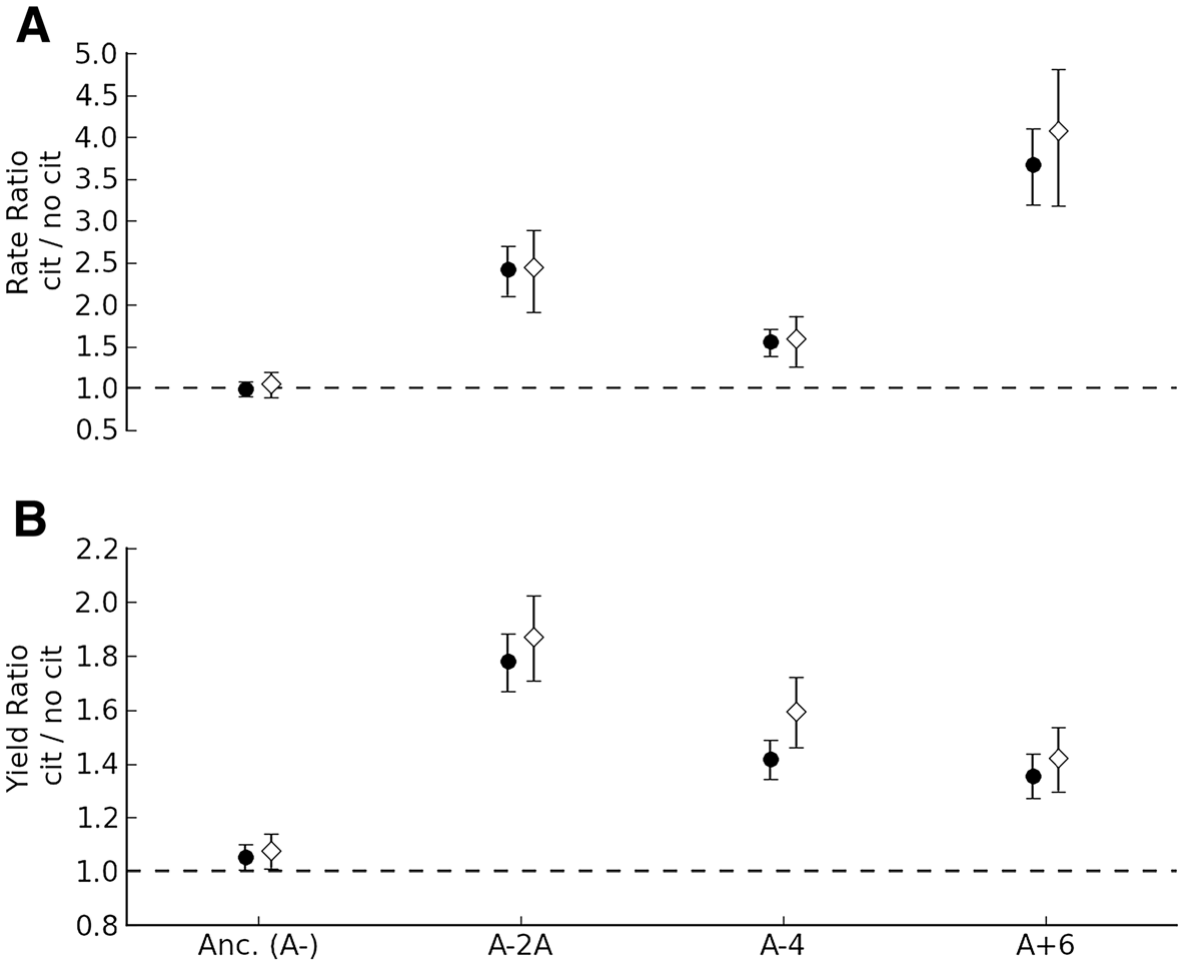
**Dependence of growth on glucose is due to citrate itself rather than the coincidental addition of sodium.** The ratio and error for growth parameters was calculated by fitting a log-linear model with the strain:media interaction terms and block as the dependent variables. Both the relative (**A**) growth rate and (**B**) yield on glucose for the three lineages that were strongly stimulated by the addition of sodium citrate (black circles) could be achieved by simply adding citric acid (white diamonds). Values represent the mean and 95% confidence intervals for the ratio of growth of that strain on glucose with the supplement to growth without.

One possible explanation for the effect of citrate could be stimulation by the addition of sodium (3.4 mM Na^+^ cations), as disodium citrate was the only source of the cation in the medium (H1). In order to test for this possible effect, we compared the stimulatory effect of disodium citrate to citric acid of the same concentration (which was pH-normalized with KOH). Both growth rates and yields with citric acid for the three citrate-stimulated evolved strains were nearly identical to those from disodium citrate (Figure 2). The only differences that were statistically distinguishable were those for yield for A-2A (P = 0.016) and A-4 (P = 0.004), which were in fact slightly higher on citric acid than on disodium citrate. This suggests that the growth phenotype we see in these evolved strains is related to citrate rather than a side-effect of sodium.

### Evolved strains are unable to utilize citrate as a carbon source

Given that citrate stimulation of glucose growth was not due to sodium, we next examined whether or not strains could directly metabolize citrate (H2). First, we asked whether any of these three citrate-stimulated strains grow with citrate as a sole carbon source. When cultured in DM containing citrate but no glucose, there was no measurable increase in OD after 24 hours (A-2A P = 0.45, A-4 P = 0.40, A+6 P = 0.72). Thus, unlike the Cit^+^ A-3 population, none of these isolates appear to be capable of growth on citrate alone.

**Figure 3:**
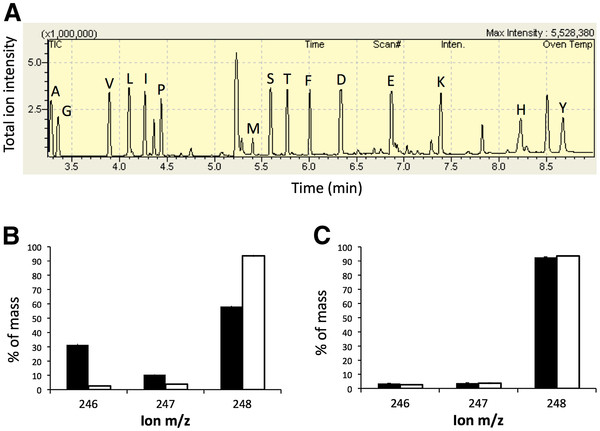
**GC-MS technique to determine if citrate was incorporated into the amino acids of biomass during glucose growth.** Amino acids from cells grown on 100% U-^13^C-glucose with or without citrate (natural abundance; ~99% ^12^C) were analyzed via GC-MS. (**A**) A representative GC-MS chromatogram of total ion intensity indicating the presence and separation of derivatized amino acids. (**B**) Mass spectra for an example fragment (from glycine) when the citrate-consuming A-3 was grown on glucose with citrate (black) or without (white), and (**C**) when A+6 was grown on glucose with and without citrate. For each amino acid, one or more characteristic mass fragments are observed whose mass distribution spectra shows the percentage of each mass relative to the total of all ions for that fragment. The example illustrates peaks 246 to 248 for the M-57 fragment of glycine, which represent the two carbons in glycine that were either both ^12^C-labeled, one ^12^C and one ^13^C, or both ^13^C-labeled, respectively. Across all amino acids, there was not a significant decrease in ^13^C-labeling from glucose except in the A-3 line where unlabeled ^12^C citrate was clearly incorporated into biomass. Error bars represent standard deviations of three biological replicates.

Although the three citrate-stimulated evolved isolates were found to still be Cit^-^, this result did not rule out possible co-metabolic use during growth on glucose. To explore this possibility, we determined whether carbon from the citrate was incorporated into biomass during growth on glucose. We grew cells in 100% U-^13^C labeled glucose at 1 mM, both with and without unlabeled (i.e., ~99% ^12^C) citrate, and looked for differential incorporation of ^13^C into the amino acids of the growing cells under these two conditions. Using gas chromatography–mass spectrometry (GC-MS), we compared the mass distribution vectors for fragments of derivatized amino acids from the cultures (Figure 3). Since CO_2_-utilizing reactions represent a source of naturally occurring carbon, it is expected to observe less than 100% ^13^C in amino acid fragments. If citrate were co-utilized it would enter directly into the citric acid cycle and be incorporated into biomass, which would dilute the incorporation of ^13^C from glucose into amino acids. We compared the peaks corresponding to fully-labeled fragments using a Wilcoxon signed-rank test. For the Cit^+^ A-3 population, there was a significant difference in incorporated label in the presence of unlabeled citrate (P = 0.0006), but for the other citrate-dependent strains there was no significant difference (A-2A: P = 0.33, A-4: P = 0.73, A+6: P = 0.47) (Figure 3B-C). These data confirm that these three strains were not utilizing citrate as a carbon source to any measurable extent.

### Citrate dependence is related to its role as an iron chelator

**Figure 4:**
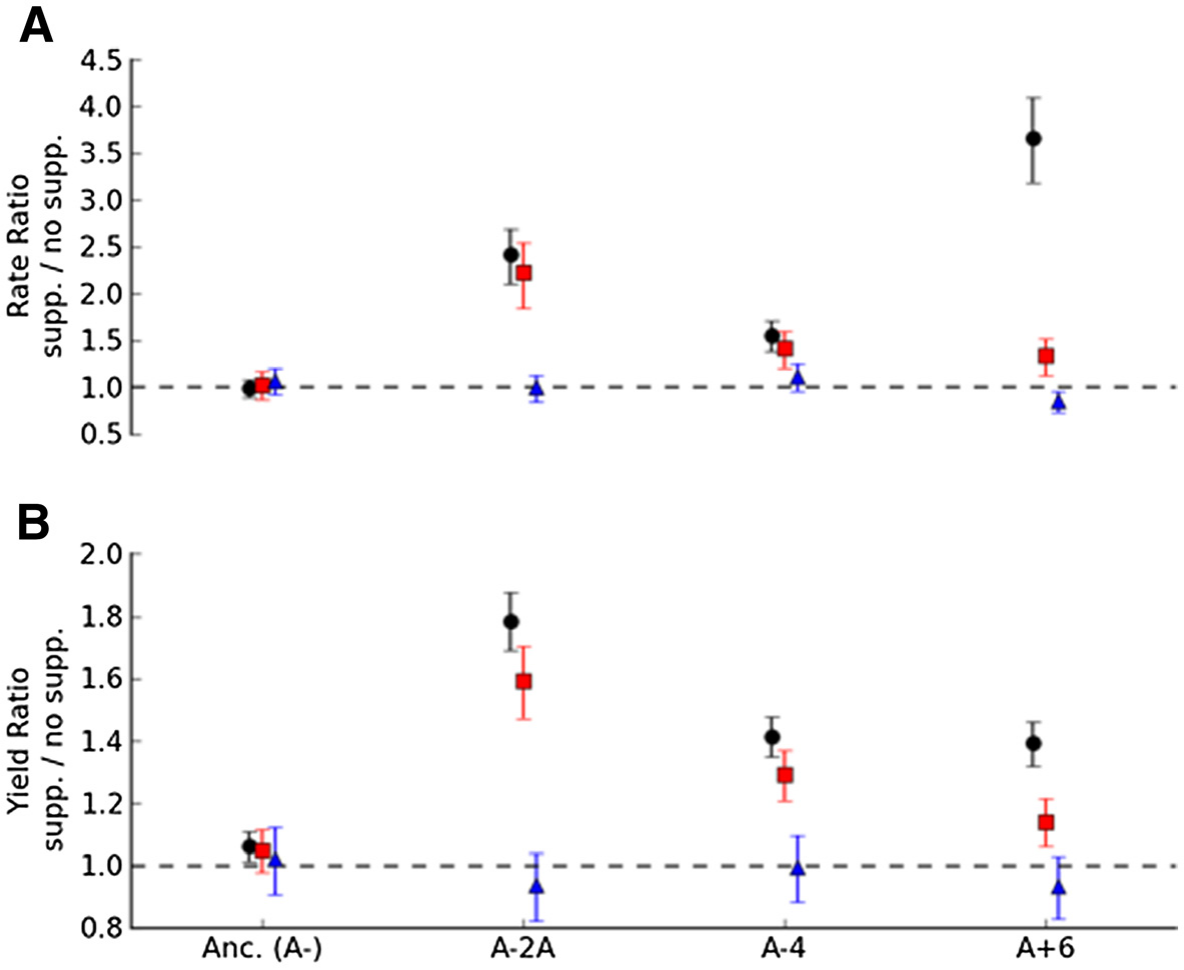
**Direct addition of iron, but not spent medium, stimulates growth on glucose for citrate-dependent evolved lineages.** The ratio and error for growth parameters was calculated by fitting a log-linear model with the strain:media interaction terms and block as the dependent variables. Both the relative (**A**) growth rate and (**B**) yield of the three strains from populations stimulated by sodium citrate (black circles) were at least partially enhanced by direct addition of 10 μM Fe (II) (as ammonium iron sulfate, red squares). In contrast, growth in spent media re-supplemented with glucose (blue triangles) did not stimulate growth. Values represent the mean and 95% confidence intervals for the ratio of growth of that strain on glucose with the supplement to growth without.

Having rejected hypotheses H0-H2 for the three citrate-dependent strains, we tested the remaining hypothesis that the effect of citrate is related to its role as a chelator in iron acquisition (H3). *E. coli* can take up ferrous iron with the Feo and Efe transporters without the involvement of citrate [25]. We first tested whether providing sufficient iron (as ferrous iron to 10 μM) in the absence of citrate would enhance growth on glucose of the three citrate-dependent evolved isolates (Figure 4). For one of the citrate-dependent strains, A-4, the iron greatly increased the growth rate and yield to values indistinguishable from that achieved via citrate (P = 0.06 and P = 0.11, respectively). A similar effect was seen for A-2A, where iron increased growth rate and yield to ~90% that achieved by citrate, a small, but significant difference (P = 0.008 and P = 5.6x10^-5^, respectively). For the third strain, A+6, the direct addition of iron significantly increased rate (P = 5.6x10^-8^) and yield (P = 0.0008), but this increase amounted to only ~40% of the yield increase and ~13% of the rate increase seen with the addition of citrate. 

One possible explanation for the evolved strains’ acquired dependence upon citrate would be a defect in either production or uptake of iron chelating siderophores. To test whether the citrate-dependent strains had become deficient in their ability to secrete siderophores, we grew the three citrate-dependent evolved isolates in media without citrate that had been conditioned by the growth of the ancestral REL606 and then re-supplemented with glucose. Unlike what has been observed previously for siderophore-defective strains [32,33], none of the citrate-dependent strains grew faster or to a higher final yield on the spent media than on DM without citrate (at an 0.05 level, Welch’s 1-tail T test) (Figure 4). This argues against defects in siderophore production as the sole cause of citrate dependency, but does not rule out the possibility that it is related to the ability to transport iron-siderophore complexes.

## Discussion

Here we report that by 50,000 generations, three strains from the 11 LTEE populations not previously shown to be dependent on citrate now grow on glucose in a manner that is strongly dependent on the presence of citrate. Thus, eight of the populations have retained the ancestral phenotype of marginal - if any - stimulation by citrate (H0 above) and one has famously become Cit^+^ and can readily grow on it as a sole carbon and energy source (H2). For the remaining three populations whose glucose-growth was substantially enhanced by citrate, our experiments allowed us to reject both the hypothesis that this stimulation was due to a side-effect of adding sodium (H1) as well as the hypothesis that it was due to co-metabolism during glucose growth (H2). The remaining hypothesis – citrate as an iron chelator (H3)– appears to best explain this phenotype, as direct addition of iron in the absence of citrate stimulated glucose growth.

What physiological mechanism may have caused this evolved dependence on citrate? The ability of ferrous iron to largely restore the phenotype of rapid growth on glucose is the strongest evidence for a loss of function in metal acquisition. The primary iron acquisition system for *E. coli* under aerobic conditions is via siderophores [25]. The fact that citrate-dependent strains did not benefit from growth on media conditioned by their citrate-independent ancestor, however, suggests that their dependence was unlikely to be due to an inability to produce or secrete siderophores. The most plausible explanation for their citrate-dependence would therefore be mutations involved in siderophore uptake. Since strains A-2A and A-4 were almost completely rescued by ferrous iron, they appeared to have no defects in their systems for direct iron uptake. On the other hand, A+6 was only mildly stimulated by ferrous iron, which would suggest this strain either has additional mutations in genes related to direct ferrous iron transport, defects in global regulators that stimulate up-regulation of iron-acquisition genes under iron starvation [24], and/or another non-iron-related dependency on the presence of citrate. Given the large number of genes involved in iron transport (the ferric uptake regulon alone consists of ~30 iron-transport-related genes [34]), the most useful first step for answering these questions will come from analysis of candidate causal mutations from the genome sequences from these lineages, which are in progress (personal communication, R.E. Lenski & D. Schneider).

At this point, it is not possible to conclude whether the new citrate dependence during growth on glucose is the result of adaptation or the neutral accumulation of mutations. Previous experiments in the LTEE have suggested that the predominant mechanism for tradeoffs in the evolved strains has been antagonistic pleiotropy, and not the accumulation of mutations [5]. However, since this initial study, 30,000 more generations have passed, and many more mutations have occurred. In the genome sequence published for the A-1 lineage, for example, there were only 29 nucleotide substitutions after the first 20,000 generations, but 598 more accumulated in the subsequent 20,000 generations. This striking increase in mutation rate was due to acquiring a mutator phenotype during the latter period [13]. In general, the respective impacts of mutation accumulation versus antagonistic pleiotropy should be revisited given the increased mutation rate in many of the strains, and the amount of time that has passed.

Though definitive statements cannot be made, mutation accumulation seems like a plausible cause of the observed acquisition of citrate dependence. Iron transport genes were presumably superfluous in the constant presence of high levels of citrate for 50,000 generations. Additionally, the large number of genes involved in iron uptake would provide a reasonably large mutational target. Finally, all three of the strains that show strong citrate dependence during growth on glucose are amongst those that have also acquired a mutator phenotype; mutations to *uvrD*, *mutL*, and *mutS* have increased their mutation rates 100–1000 fold [5,35]. This result in itself is not statistically significant, as 6 of the parallel populations have acquired this phenotype by 50,000 generations, so the probability by chance that all three citrate-dependent strains are mutators is 0.1. The fact that they are all mutators, however, is consistent with the hypothesis that mutation accumulation drove the observed phenotypic changes. As with the above speculation about the identity of the mutational targets leading to citrate-dependency, the selective mechanism leading to their incorporation will require identification and introduction into the appropriate backgrounds to test the fitness effects of the dependency-inducing mutations.

## Conclusions

We have uncovered a second form of interaction with citrate that occurred during the 50,000 generations of the LTEE: three of the 12 Cit^-^ strains we examined now required its presence as an iron chelator for maximal growth on glucose. This interaction was due to an acquired dependence on citrate to carry out a function that populations have lost, which contrasts with the gain of novel function observed in the Cit^+^ A-3 population. Unlike the strong selection for the ability of A-3 to catabolize citrate, selection was likely either weak or absent in the loss of citrate-independent glucose performance. In this regard, the constant environment of the LTEE was perhaps not unlike that encountered along the evolutionary path to intracellular symbionts, whereby specialization emerges not so much from gain of new traits as from the persistent loss of traits rendered unnecessary.

A final subtext for considering the emergence of citrate-dependence during glucose growth is that subtle decisions in the design of evolution experiments can have unpredictable impacts. By addressing one complication in experimental design, one can unknowingly create new adaptive opportunities. Citrate was present in DM media from the start of the experiment despite a quite small, marginal difference between growth of the ancestor in its presence or absence. This seemingly insignificant detail – combined with tens of thousands of generations of adaptation – has led to unexpected phenomena that range from the incredible acquisition of aerobic citrate metabolism, to the dependence on citrate as an iron chelator.

## Methods

### Strains and LTEE conditions

*Escherichia coli* B isolates were obtained from the Lenski LTEE [11] after 50,000 generations. Briefly, 12 populations of *E. coli* were founded with either the arabinose-negative strain REL606 (populations A-1 to A-6) or the arabinose-positive derivative, REL607 (A+1 to A+6). These have been evolved since 1988 in 50 mL flasks containing 10 mL of modified Davis-Mingioli (DM) minimal media [36] (which we refer to as DM media) with 139 μM glucose (25 mg/L) as a growth substrate. One liter of DM consists of 7 g potassium phosphate (dibasic trihydrate), 2 g potassium phosphate (monobasic anhydrous), 1 g ammonium sulfate, 0.5 g disodium citrate, 1 mL 10% magnesium sulfate, 1 mL 0.2% thiamine (vitamin B1), and a carbon source- here glucose. These populations have been cultured at 37°C while shaking at 120 rpm, and have been transferred daily via 1:100 dilutions (~6.64 net doublings per day).

The isolates analyzed in this experiment consisted of the ancestral lines REL606 and REL607, as well as the ‘A’ clone frozen at 50,000 generations from the 12 populations (A-1A: REL11330, A-2A: REL11333, A-3: REL11364, A-4A: REL11336, A-5A: REL11339, A-6A: REL11389, A+1A: REL11392, A+2A: REL11342, A+3A: REL 11345, A+4A: REL11348, A + 5A: REL11367, A+6A: REL11370). The A-2A clone is from the ‘large’ lineage that has coexisted with a cross-feeding ‘small’ lineage for tens of thousands of generations [31]. We therefore also examined an A-2C ‘small’ clone (REL11335).

Before growth and GC-MS experiments, acclimation cultures were inoculated from freezer stocks and grown for 24 hours in DM 1 mM glucose without citrate.

### Growth rate and yield experiments

To measure growth rate and yield, cultures were grown in 50 mL flasks containing 10 mL of DM minimal media (the same media as the evolution experiment) with 1 mM glucose. In order to avoid extremely low optical densities, growth assays were conducted at 1 mM, which is higher than the 25 mg/L = 0.14 mM that was used in the “DM_25_” medium of the evolution experiment. It is, however, closer to the concentration of glucose in the evolutionary environment than either the 250 mg/L or 1000 mg/L concentrations that had previously been used for physiological assays on these strains [13,30,37]. DM + citrate media contained disodium citrate at a concentration of 1.7 mM, as normal. DM + iron was supplemented with ammonium iron sulfate, mixed fresh the day of the experiment, to 10 μM of Fe (II). Ammonium is already present in DM (as ammonium sulfate) at a concentration of 7.6 mM, so 10 μM addition in iron sulfate does not meaningfully change its concentration. DM + citric acid was DM supplemented with 1.7 mM citric acid and pH balanced with KOH. Spent media was produced by growing the ancestral strain REL606 in DM 1 mM glucose without citrate for 24 hours, centrifuging to remove the majority of the cells, and then filtering with a 0.2 μm filter to sterilize. Citrate was omitted in order to ensure that it did not suppress siderophore production. This spent media was supplemented to 1 mM glucose with filter-sterilized 10% glucose solution, and compared to DM without citrate identically supplemented with glucose.

For growth rate experiments, OD_600_ was measured on a BioRad SmartSpec Plus (Philadelphia, PA) spectrophotometer every hour until there was a detectable increase in OD, then approximately every 30 minutes until stationary phase was reached. Yield was measured as OD_600_ after 24 hours. Growth rate was calculated with Growth Curve Fitter, an in-house software package that fits exponential curves using points in the log-linear range of observations (N.F. Delaney & CJM, unpublished). To determine growth rates, we subtracted the blank (optical density at t = 0), and fitted all points after a change in OD was measurable until the point before stationary phase was reached (4–6 points). The exception was for the Cit^+^ A-3 strain, for which the growth rate was fitted for growth on only the glucose portion of the diauxic growth curve. Between 4 biological replicates (for citrate-insensitive strains) and 19 (for the ancestor) were run for each condition. The statistical difference between growth parameters for different conditions was calculated directly from the data using Welch’s T test. In order to plot the relationship between the data in an intuitive way as a ratio, the ratio and error for growth parameters was calculated by fitting a log-linear model with the strain:media interaction terms and block as the dependent variables using R (R-Project software).

### GC-MS analysis of incorporation of carbon from glucose versus citrate

Incorporation of ^13^C was measured as previously described [38]. Strains were grown with or without citrate in 100 mL of DM media with 1 mM U-^13^C glucose (Cambridge Isotope Laboratories, Andover, MA). At stationary phase, all cells were pelleted from the media, hydrolyzed overnight in 6 M HCl, and dried. The dry cell material was then derivatized for an hour at 85°C with 40 μL each of dimethylformamide (DMF) and N-tert-butyldimethylsilyl-N-methyltrifluoroacetamide with 1% tert-butyldimethyl-chlorosilane (TBDMSTFA). The derivatized cell material was injected into a Shimadzu QP2010 GC-MS (Columbia, MD). The injection source was 230°C. The oven was held at 160°C for 1 min, ramped to 310°C at 20°C min^-1^, and finally held at 310°C for 0.5 min. The flow rate was 1 mL min^-1^ and the split was 10. The column was a 30 m Rx1-1 ms (Restek, Bellefonte, PA). Three biological replicates were run for each isolate (except A-2A, for which one run failed).

In all samples the percent of each amino acid that was uniformly labeled with ^13^C molecules was determined using FiatFlux qualitycheck [38]. For each amino acid fragment that was detected in all biological replicates, the difference in average percent of uniformly labeled fragment was determined for samples grown with and without citrate. Finally we used a non-parametric Wilcoxon signed-rank test to determine whether there was a significant difference in labeling between treatments.

## Abbreviations

LTEE: Lenski Long-term evolution experiment; DM: Davis-Mingioli minimal media; GC-MS: Gas chromatrography-mass spectrometry; Cit: Citrate; OD: Optical density.

## Competing interests

The authors declare that they have no competing interests.

## Authors’ contributions

NL, WRH and CJM conceived and designed the study. NL and WRH conducted the experiments and analyzed data. NL, WRH, and CJM wrote the manuscript. All authors read and approved the final manuscript.
